# Fluoroquinolone-Resistant *Mycobacterium tuberculosis*, Pakistan, 2005–2009

**DOI:** 10.3201/eid1703.100957

**Published:** 2011-03

**Authors:** Kauser Jabeen, Sadia Shakoor, Shazia Chishti, Afsheen Ayaz, Rumina Hasan

**Affiliations:** Author affiliation: Aga Khan University Hospital, Karachi, Pakistan

**Keywords:** Fluoroquinolone, drug resistance, Pakistan, tuberculosis and other mycobacteria, bacteria, letter

**To the Editor:** Pakistan is 1 of 22 countries listed by the World Health Organization (WHO) as having a high incidence of tuberculosis (TB). We recently reported an increase in rates of multidrug-resistant (MDR) TB with emergence of extensively drug-resistant TB (*1*). Fluoroquinolone resistance is associated with worse outcome in patients with MDR TB (*2*). Recent evidence suggests emergence and increasing incidence of fluoroquinolone-resistant *Mycobacterium tuberculosis* from several countries, particularly in MDR strains (*3*). We present data from a tertiary care referral center laboratory in Pakistan to assess fluoroquinolone resistance in MDR TB strains during 2005–2009.

The Aga Khan University Hospital and its clinical laboratory have been accredited by the Joint Commission of International Accreditation and designated as a technical partner of the National TB Program. *M. tuberculosis* susceptibility testing is also periodically validated by the WHO Supranational Reference Laboratory network. The microbiology laboratory serves different cities across Pakistan with ≈180 peripheral collection units. Specimens for TB cultures are requested by physicians and received through passive collection and thus are not restricted to programmed surveys. All specimens received at each of the collection units are sent to the central laboratory in Karachi for culture and drug susceptibility testing (DST). Specimens reach the main laboratory within 24 hours after receipt.

During the past 4 years, the laboratory has received 12,000–15,000 specimens annually for *M. tuberculosis* culture; positivity rate has been 15%–20%. Culture and DST are performed at the laboratory in accordance with Clinical Laboratory Standards Institute and WHO recommendations, as described (*4*). During 2005–2008, fluoroquinolone susceptibilities for all MDR and polydrug-resistant isolates were determined by using ciprofloxacin (2 μg/mL). From 2009 onwards, fluoroquinolone susceptibilities were determined by using ofloxacin (2 μg/mL), and second-line DST was performed for all *M. tuberculosis* isolates.

During 2005–2009, a total of 11,263 cultures were reported positive for *M. tuberculosis*. Of these, 34.4% were MDR, and 50.1% were sensitive to all 4 first-line agents (isoniazid, rifampin, pyrazinamide, ethambutol). Because of inconsistencies in testing criteria for fluoroquinolones (fluoroquinolone testing being conducted primarily for MDR cases during 2005–2008), the overall fluoroquinolone-resistance rate could not be determined. However, for MDR strains, fluoroquinolone susceptibilities were consistently determined, and resistance rates increased from 17.41% in 2005 to 42.92% in 2009 (p<0.001, by χ^2^ test for trend analysis) (Table).

A progressive increase in fluoroquinolone use and its association with increase in resistance against organisms other than *M. tuberculosis* have been reported from Pakistan (*5*). We report a progressive increase in fluoroquinolone resistance rate in MDR *M. tuberculosis* isolates during a 5-year period. This finding is consistent with those of several studies reporting fluoroquinolone resistance from the region. Agrawal et al. have recently reported an exponential increase in fluoroquinolone resistance in India from 3% in 1996 to 35% in 2004 (*6*). A significant increase in fluoroquinolone resistance from 7.7% to 20% in MDR TB was also reported from Taiwan (*7*); the authors correlated this finding with the inappropriate use of fluoroquinolones for managing TB rather than with fluoroquinolone misuse in the community. Another study from the United States and Canada reported 4.1% fluoroquinolone resistance in MDR TB strains (*8*).

In addition to detecting increasing fluoroquinolone resistance in MDR isolates, we have also detected fluoroquinolone resistance in non-MDR, polydrug-resistant *M. tuberculosis* isolates. Moreover, in 2009, a total of 3.1% of isolates susceptible to all first-line agents were fluoroquinolone resistant.

Although our dataset includes samples from throughout Pakistan, sampling limitations prevent us from deriving definite conclusions and generalizing results to the entire population of the country. A referral bias attributable to passive sampling exists because cases referred to our laboratory tend to be more complicated. Moreover, treatment history was not available for patients in our dataset; therefore, increased fluoroquinolone resistance could not be correlated with prior fluoroquinolone use. However, our findings have implications for therapy with fluoroquinolones for TB and other infections.

Fluoroquinolones are freely available as over-the-counter medications to the general population (*9*), which creates potential for misuse of fluoroquinolones by the general population for TB and several other infections, such as enteric fever and genitourinary infections. Furthermore, because national guidelines for treating enteric fever and genitourinary infections do not exist, these drugs are also overprescribed by physicians. We propose control of over-the-counter availability of fluoroquinolones and judicious use of this class of drugs by physicians to prevent further escalation in resistance rates in Pakistan.

**Table:** Resistance patterns and fluoroquinolone resistance rates of *Mycobacterium tuberculosis* isolates from the Aga Khan University Hospital laboratory, Karachi, Pakistan, 2005–2009*

Year and isolates	Sensitive†	Multidrug resistant	Extensively drug resistant	Other resistance patterns‡	Total
2005					
Total	773	643	5	361	1,782
Fluoroquinolone resistant	NT	112 (17.41)	5 (100)	6 (1.66)	NA
2006					
Total	949	728	11	195	1,883
Fluoroquinolone resistant	NT	128 (17.58)	11 (100)	7 (3.58)	NA
2007					
Total	1,054	782	17	158	2,011
Fluoroquinolone resistant	NT	163 (20.84)	17 (100)	8 (5.06)	NA
2008					
Total	1,305	991	32	256	2,584
Fluoroquinolone resistant	NT	351 (35.41)	32 (100)	17 (6.64)	NA
2009					
Total	1,560	1,181	53	209	3,003
Fluoroquinolone resistant	48 (3.07)	507 (42.92)	53 (100)	23 (11)	631 (21.01)
p value§	NA	<0.001	NA	<0.001	NA
Total	5,641	4,325	118	1,179	11,263


*Values are no. (%) isolates in that category, except p values. NT, not tested; NA, not applicable. †To isoniazid, rifampin, pyrazinamide, and ethambutol. ‡Other resistance patterns include isoniazid-monoresistant and polydrug-resistant isolates. §χ^2^ trend analysis.
